# Inhibition of Adhesion, Proliferation, and Invasion of Primary Endometriosis and Endometrial Stromal and Ovarian Carcinoma Cells by a Nonhyaluronan Adhesion Barrier Gel

**DOI:** 10.1155/2015/450468

**Published:** 2015-02-15

**Authors:** Stefan P. Renner, Pamela L. Strissel, Matthias W. Beckmann, Johannes Lermann, Stefanie Burghaus, Janina Hackl, Peter A. Fasching, Reiner Strick

**Affiliations:** University Clinic Erlangen, Department of Gynaecology and Obstetrics, University Endometriosis Centre Franconia, Laboratory for Molecular Medicine, Friedrich-Alexander University Erlangen-Nuremberg (FAU), 91054 Erlangen, Germany

## Abstract

Endometriosis is a chronic disease of women in the reproductive age, defined as endometrial cells growing outside of the uterine cavity and associated with relapses. Relapses are hypothesized to correlate with incomplete surgical excision or result from nonrandom implantation of new endometrial implants in adjacent peritoneum. Thus, surgical excision could lead to free endometriotic cells or tissue residues, which readhere, grow, and invade into recurrent lesions. Barrier agents are frequently used to prevent postoperative adhesions. We tested if the absorbable cell adhesion barrier gel Intercoat consisting of polyethylene oxide and sodium carboxymethyl cellulose could inhibit cellular adhesion, proliferation, and invasion of primary endometriosis and endometrial cells. Due to an association of endometriosis with ovarian carcinoma, we tested two ovarian carcinoma cell lines. Prior to cell seeding, a drop of the barrier gel was placed in cell culture wells in order to test inhibition of adherence and proliferation or coated over a polymerized collagen gel to assay for prevention of invasion. Results showed that the barrier gel significantly inhibited cell adherence, proliferation, and invasion of endometriosis and endometrial stromal cells as well as ovarian carcinoma cells in culture. Our findings could help to prevent local cell growth/invasion and possible consequent recurrences.

## 1. Introduction

Endometriosis is a chronic disease that affects approximately 10% of women in the reproductive age and has a major impact on a patient's quality of life [1]. The main symptoms include infertility and pain [2, 3]. Endometriosis is defined as endometrial-like glands and stroma growing outside the uterine cavity, especially in the peritoneum affecting organs like the ovaries, fallopian tubes, colon, and uterus [4]. Main treatment options are medical and surgical, while the combination of both in the initial stage of the disease is the preferred goal. Though surgical options have improved over the years and removal of lesions in the peritoneal cavity can be achieved with minimal invasive techniques, symptom recurrence and requirement for reoperation appear to be progressive over time (at 1 year approximately 15%, at 5 years 36%, and by 7 years 50%) [5, 6]. After initial surgery histologically verified recurring endometriosis lesions were found to correlate near the original lesion sites, thus supporting the hypothesis that either incomplete excision occurred or endometrial tissues implanted nonrandomly [7]. In contrast, successful excision of deep infiltrating endometriosis can be achieved in over 90% of cases and is therefore considered rarely progressive and recurrent [8].

Postoperative adhesions occur in 60–90% of patients after laparoscopy, laparotomy, or abdominopelvic surgery [9, 10]. These high adhesion rates have been associated with symptoms of intestinal obstruction, diffuse chronic pelvic pain, and infertility [11]. Importantly, different barrier agents which were tested in animal models and in patients receiving different gynecological surgeries including endometriosis and ovarian carcinoma demonstrated significant reduction of adhesions (see Table 1 for summary) [12–15]. When a barrier substance is applied as a single layer to traumatized tissue this temporary barrier helps to promote the healing process. Furthermore, adhesion prevention is believed to reduce pain after surgery and increase future pregnancy rates. Studies about pregnancy rates after application of barrier substances are rare while studies about pain and recurrence are lacking [11, 16, 17]. Additionally, studies regarding the color phenotype of the lesion and the application of barrier agents in endometriosis are scarce. There is some evidence that a prophylaxis with an adhesion barrier gel is more effective in patients with red lesions than in patients with black, white, or clear lesions, implying a positive influence with the use of adhesion barrier gels on endometriosis lesions [18]. It has also been described that liquid barrier agents might have potential anti-inflammatory, antioxidant, and antitumoral properties using* in vitro* models [19, 20].

In the pathogenesis of endometriosis various factors in the peritoneal environment have been proposed to regulate endometriosis growth, angiogenesis, cellular remodeling, and inflammation [21]. Additionally, hyaluronan, a normal component of the extracellular matrix, promotes cell migration, differentiation, and proliferation and is the main substance present in some antiadhesion barriers [22] (Table 1). Generally all human epithelial tumors are surrounded by stroma enriched in hyaluronan and its amount represents a strong, independent, negative predictor of patient survival, especially for breast and ovarian cancers [22, 23]. Depending upon the cell lines (e.g., colorectal, colon, and ovarian) or animal models used, both positive and negative cellular effects of hyaluronan containing barriers or the pure substance alone have been noted* in vitro* and* in vivo* [24–27]. There are also a few retrospective studies involving cancer patients where barrier gels containing hyaluronan were implemented during surgery and showed no significant differences in patient survival compared to controls [28] (Table 1).

Both eutopic stromal endometrial (Eu-ESC) and ectopic endometriosis stromal cells (Ec-ESC) demonstrate migration and invasive properties, with a resemblance to cancer cells [29]. As initially proposed by Sampson, menstrual blood consisting of free eutopic endometrial cells or tissue fragments undergo retrograde efflux into the peritoneal cavity, which can result in pelvic or ovarian endometriosis as well as adenomyosis [30]. Interestingly, endometriosis has been positively associated with ovarian carcinoma, especially the clear cell, endometrioid, and low grade serous subtypes [31, 32]. Similar to invasion models of cancer cells, cell culture systems studying migration and invasion of endometriosis and endometrial cells have implemented Matrigel. For example, SV40 transformed Ec-ESC cell lines were established and exhibited the same invasive and cell phenotypic characteristics of explants [33]. Other studies comparing Eu-ESC with Ec-ESC showed higher invasion with Matrigel for endometriosis cells [34, 35]. On the other hand Ec-ESC showed less migration/invasion through fibronectin/collagen coated chambers compared to Eu-ESC, due to increased contractile properties [36]. Finally, in a mouse model Ec-ESC could migrate back to the uterine endometrium and exhibited characteristics of an EMT by gene expression analysis [37].

In the present study, we used a common cell barrier called Intercoat gel containing polyethylene oxide (PEO) and sodium carboxymethyl cellulose (CMC) stabilised with calcium to test if this compound exhibited inhibitory effects against cell adhesion, proliferation, and invasion of primary Eu-ESC with Ec-ESC lines in culture. Due to an association of endometriosis with ovarian carcinoma and the fact that ovarian carcinoma exhibits increased proliferation and invasion properties we also tested the barrier gel with one endometrioid and one clear cell ovarian carcinoma cell line using the same methodologies.

## 2. Materials and Methods

Endometriosis tissue samples from the peritoneal wall were from three patients (mean age = 29.3 years) with American Society of Reproductive Medicine (ASRM) scores = 2. Patients were seen due to the presence of pelvic pain. Nondiseased endometrial tissue samples were from three nonmatched patients with endometriosis (mean age = 29.6 years). Tissue probes were from patients at a first diagnosis. All patients had menstrual cycles and no other diseases or prior treatment. In addition, one cell line TOV112D (ATCC: CRL-11731) from an endometrioid ovarian carcinoma and the TOV-21G cell line (ATCC: CRL-11730) from a clear cell ovarian carcinoma were chosen. All patients with endometriosis were scheduled for surgery independently of the study and were informed about the study prior to surgery. All tissue samples were sent to pathology for verification where only additional tissue from the same location was used for the present study. The study was approved by the Ethic Committee of the University Erlangen-Nuremberg (number 2567).

### 2.1. Isolation of Primary Ec-ESC and Eu-ESC Cells

Ec-ESC cell was isolated from lesions according to Kao et al. with some modifications [35]. Briefly the endometriosis lesion was washed 2 times with PBS and minced into small pieces. The pieces of the endometriosis lesion were then treated with 1 mg/mL collagenase type IA (Sigma-Aldrich, Munich, Germany) in RPMI media without serum for 1.5 hr at 37°C and 5% CO_2_ on a rotator. The separation of the cells was monitored every 15 min. A further dissociation of endometriosis cells was performed in the presence of 0.015% of Trypsin (Life Tech., Darmstadt, Germany) and 0.6 U/mL Dispase (Roche Applied Sci., Mannheim, Germany) for 30 min at 37°C and 5% CO_2_. The cells were then filtered through a 100 *μ*m filter (BD Biosciences, Heidelberg, Germany) in order to clear any fibrotic tissue pieces. Single cells were counted and seeded in RPMI with 5% FCS initially in 3.5 cm culture dishes and expanded for growth. The same methodology above was performed using endometrial tissue; however the use of a 100 *μ*m filter was not needed.

In order to identify the epithelial (cytokeratin-7 positive) or stromal phenotype (vimentin positive) of the isolated cells immunofluorescence was performed. Cells were grown on FCS coated coverslips for 3 days and fixed for 15 min with 4% p-formaldehyde, washed 3 times with 1x PBS, and then permeabilized with 0.1% Triton-X100 (Sigma-Aldrich) for 10 min at room temperature (RT). Cells were washed with 1x PBS and then blocked with 5% horse serum and 1% BSA (Sigma-Aldrich). An overnight incubation was performed at 4°C with antivimentin conjugated with FITC (Fitzgerald, Poggensee, Germany) or anticytokeratin-7 (Sigma-Aldrich) (each 1 : 100). The next day cells were washed 3 times with 1x PBS, and a secondary antibody goat anti-mouse PE (1 : 500) (Antibodies-Online) was used to detect cytokeratin-7. In addition, DAPI (Sigma-Aldrich) staining was implemented to detect nuclei. In the present study only vimentin positive Ec-ESC and Eu-ESC cells were used.

### 2.2. Adhesion and Proliferation Analysis

For adhesion and proliferation assays, 6-well culture dishes (3.5 cm/well) (BD, Heidelberg, Germany) were coated with and without 1 mL/well of an absorbable adhesion prevention barrier gel, Intercoat, purchased in the standard package (two 20 mL syringes) from Johnson and Johnson (Ethicon Intercoat). This barrier gel chemically represents a combination of polyethylene oxide (PEO) and sodium carboxymethyl cellulose (CMC), which is stabilised with calcium and then brought to isotonic conditions with NaCl. To quantitate if cells could adhere and proliferate, 50,000 of Ec-ESC, Eu-ESC or 50,000 cells of each ovarian carcinoma cell line were seeded per well in media (RPMI with 10% FCS) either without or on the Intercoat cellular barrier. Experiments were performed in triplicate for Ec-ESC, Eu-ESC and duplicate for ovarian cell lines. Following six days of culture, all cells were collected and counted using a Luna-TM automated cell counter (Biozym, Germany) to determine the total number of live versus dead cells (Trypan blue positive). A second experiment was performed where a drop (500 *μ*L) of Intercoat cellular barrier gel in the form of a circle was placed in the middle of a 3.5 cm well. Ec-ESC, Eu-ESC, or each ovarian carcinoma cell line was resuspended in RPMI media and 10% FCS, added to the well, and then monitored microscopically and photographed daily to test how long the cells were blocked for adherence and growth. Experiments were performed in triplicate.

### 2.3. Invasion Analysis

Cell invasion analysis was performed according to Wacker et al. [38]. Calf skin type I collagen G (Serva Electrophoresis GmbH, Heidelberg, Germany) and rat tail type I collagen R (Biochrom AG, Berlin, Germany) were mixed at a ratio of 1 : 1 plus 0.1 volume of sodium bicarbonate (23 mg/mL), 0.1 volume of 10x RPMI, and then the solution was neutralized with sodium hydroxide. An aliquot of 1.2 mL was added to each well of a six-well plate and polymerized (0.5 cm bed) at 37°C for 1 hr. Sterile Intercoat gel (1.0 mL) was coated over the surface of the collagen for 30 min at RT before cell seeding. Ec-ESC, Eu-ESC, and ovarian carcinoma cells (100,000 cells per well) were added in RPMI media plus 10% FCS to the collagen alone or on top of an Intercoat gel/collagen surface. After 6 days invaded cells were detected by focusing down below the surface of the collagen matrix and 20 optical fields per well were counted by two researchers. Floating cells were also assessed by focusing above the collagen surface in the media. All cell lines were performed in duplicate.

### 2.4. Statistical Analyses

The nonparametric Mann-Whitney  *U*  test for independent samples was performed using IBM SPSS Statistics 19 (IBM, Germany). For all tests a *P* value < 0.05 was considered statistically significant. For each mean value, a standard error of the mean (s.e.m.) was calculated using IBM SPSS Statistics 19.

## 3. Results

**Figure 1:**
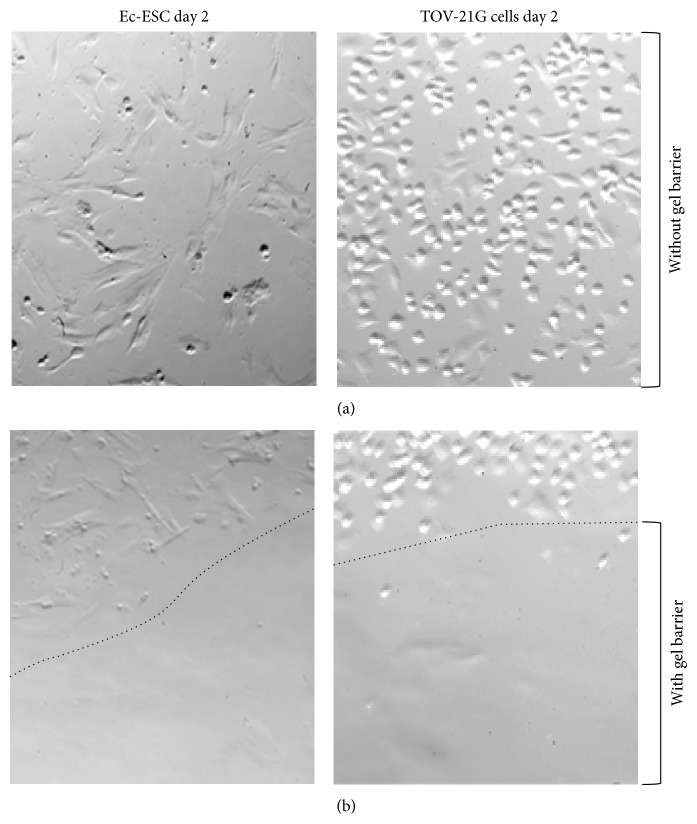
Microscopic analysis on day 2 showing inhibition of cell adherence and growth around or within a drop of the barrier gel. (a) Showing normal mitotic growth of both Ec-ESC and the ovarian carcinoma cell line TOV-21G 48 hr after setup with no barrier substance. (b) Showing a visual direct inhibition of adherence and growth around (dotted line) or within a drop of the barrier gel.

**Figure 2:**
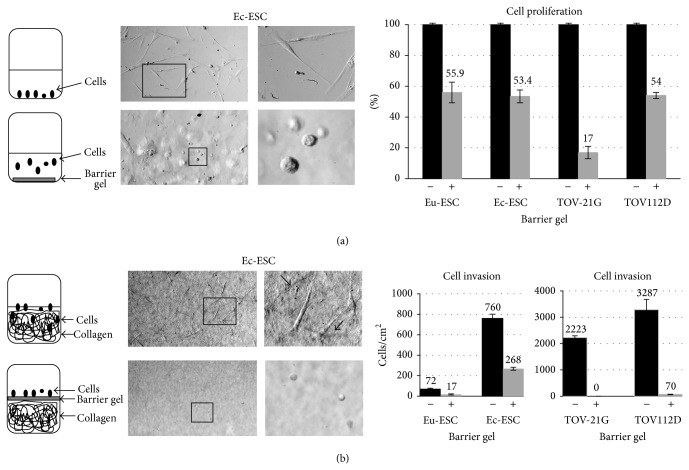
Microscopic analysis on day 6 showing inhibition of cellular adherence, proliferation, and invasion in the presence (+) or absence (−) of a barrier gel. (a) Left panels show schematic drawings of the overall cell culture experimental design and outcome of results. Top microscope pictures show normal mitotic growth of Ec-ESC without the presence of a barrier substance with the boxed region representing a magnification (right picture). Bottom microscopic pictures show a region, where cells were inhibited in adherence when the barrier gel was entirely coated on the bottom of a 35 mm tissue culture well. The boxed region shows a magnification (right picture). Note that Ec-ESC cells are rounded and floating. The presence of the barrier gel also led to lower cellular proliferation as represented in the graph to the right. Graph shows percentage of cellular growth after 6 days of culture of Eu-ESC and Ec-ESC and both ovarian carcinoma cell lines, when the barrier substance was entirely coated on the bottom of a 35 mm cell culture well. Untreated cells were set to 100%; mean values (numbers above graphs) are shown from experiments of Eu-ESC and Ec-ESC (triplicates) as well as ovarian carcinoma cell lines (duplicates). Percentage values in the presence of the barrier gel were for Eu-ESC: 55.9 +/− 0.67; Ec-ESC: 53.4 +/− 4.2; TOV-21G: 17 +/− 4.0; TOV112D: 54 +/− 2.0. (b) Left panels show schematic drawings of the overall cell invasion experimental design and outcome of results. Top microscope pictures show the collagen surface without a barrier substance and Ec-ESC adhered cells, which invaded collagen. The right picture represents a magnification of the left box. The arrows indicate Ec-ESC, which invaded below the collagen surface. The bottom panel represents the results of Ec-ESC in the presence of a barrier gel entirely coated over the collagen surface. A region is shown, where Ec-ESC cells were inhibited in adherence and invasion and thus were rounded and floating in media above the collagen surface. The boxed region shows a magnification (right picture). Right two graphs show the amount of invaded cells into collagen per cm^2^ after 6 days with or without the barrier gel coated on the collagen surface. Mean values of invaded cells are shown in the graphs in the presence (+) or absence (−) of the barrier gel: Eu-ESC: 72 +/− 5.6 (−); Eu-ESC: 17 +/− 6.3 (+); Ec-ESC: 760 +/− 40.7 (−); Ec-ESC: 268 +/− 14.6 (+); TOV-21G: 2,223 +/− 74.2 (−); TOV-21G: 0 +/− 0.0 (+); TOV112D: 3,287 +/− 389.9 (−); TOV112D: 70 +/− 11.2 (+).

In order to test the influence of polyethylene oxide and sodium carboxymethyl cellulose (Intercoat) barrier gel on cellular adhesion, proliferation, and invasion we initiated* in vitro* cell culture studies with eight different cell lines. These included three Ec-ESC and three Eu-ESC cell lines as well as two ovarian carcinoma cell lines. Microscopic inspection of all cell lines demonstrated that the barrier gel inhibited adherence or growth directly around or within the drop of the gel (Figure 1 and data not shown). In contrast, all cells not in contact with the gel showed normal morphology, attachment, and mitotic growth (Figure 1 and data not shown). A daily microscopic examination of the barrier gel drop/cellular border revealed that the barrier gel remained intact as a drop and attached to the culture dish for approximately three days, thereafter, lifting off and partly solubilizing. In another experiment, when the barrier gel was coated on the bottom of a 35 mm cell culture well, in contrast to untreated controls all cell lines exhibited more floating round cells, which as we interpreted were inhibited in their ability to adhere to cell culture wells (Figure 2(a)). After a total of 6 days in the presence of the barrier gel the attached cells were trypsinized and the total number of live cells was measured. Results showed that compared to untreated control experiments the presence of the barrier gel inhibited proliferation of Ec-ESC (mean 47%, *P* = 0.024) and Eu-ESC cells (mean 45%, *P* = 0.023) as well as both ovarian carcinoma cell lines (TOV-21G mean 83%; TOV112D mean 46%) (Figure 2(a)).

**Figure 3:**
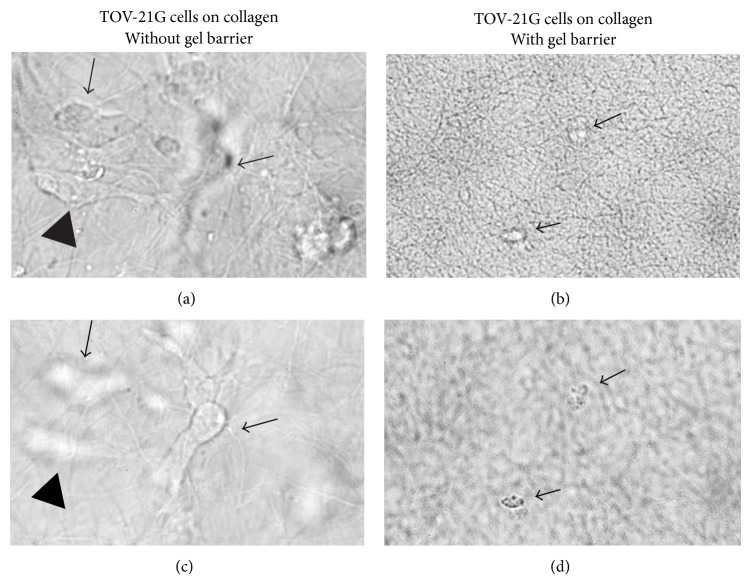
Inhibition of TOV-21G cellular invasion into a collagen matrix on day 6. (a) Top left picture shows a microscopic region focused on the collagen surface on day 6. Cells adhered to the surface are in focus (e.g., arrowhead), whereas the out of focus cells have invaded to different depths beneath the surface (arrows). After focusing the microscope beneath the surface of the collagen, the deepest invaded cell from the top picture is now visible (middle arrow and bottom left picture), whereas the other two cells are out of focus (arrowhead and arrow). Two right pictures represent a collagen surface entirely coated with the barrier gel on day 6 of cell culture. Top right picture shows a region, where no cells have adhered to the collagen surface. Arrows point to two out of focus floating cells. After focusing the microscope above the collagen surface in the media both dead cells became visible (arrows and bottom right picture).

In order to compare the invasive potential of Ec-ESC and Eu-ESC with ovarian carcinoma cells, collagen gels were polymerized in tissue culture wells. Before the wells were seeded with cells a thin layer of the barrier gel was coated on the surface of collagen gels (Figures 2(b) and 3). Importantly, over a period of 6 days we observed that, for all collagen gels coated with the barrier gel, invasion was significantly inhibited for Ec-ESC (*n* = 3) (2.8-fold, *P*: <0.0001), Eu-ESC (*n* = 3) (4.2-fold, *P*: <0.0001), and the ovarian carcinoma cell lines almost completely (Figure 3). For untreated collagen gels as predicted ovarian cancer cells showed the highest number of invaded cells below the collagen surface. For Ec-ESC an approximate 10.5-fold mean increase of invasion (*n* = 3) was found when compared to Eu-ESC (*n* = 3).

## 4. Discussion

Postoperative adhesions occur frequently after gynecological surgeries with a major impact on the patients' quality of life due to pain, infertility, and multiple operations [11, 39]. Barriers are commercially available to prevent adhesions [16] (Table 1). Endometriosis is a chronic benign disease with a high recurrence rate even after optimal surgery [5, 6]. The influence of endometriosis associated with pain and infertility rates is still under investigation and large prospective studies are needed to help further define relapses [17]. Many authors describe a relapse as histologically confirmed reoccurrence of endometriosis lesions. As some patients with endometriosis are asymptomatic all patients need to have additional surgeries although not necessarily required. An alternative definition of relapse could be the reoccurrence of symptoms. Although using this definition endometriosis would not be histologically confirmed, medical options could be chosen in some cases to prevent additional operations [6]. Presently, medical options to lower the recurrence rates in patients with endometriosis and to prevent multiple surgeries are either hormonal (i.e., oral contraceptives, progestins) or antihormonal (GnRH-Analogues). All substances show considerable side effects including malaise, headaches, and weight gain as typical side effects of hormonal therapy. Patients taking GnRH-Analogues show even more severe side effects including menopausal symptoms, for example, hot flashes, depression, and osteoporosis. All therapeutic options prevent pregnancies and thus are not reasonable for patients who are trying to conceive. Barrier substances are often applied during surgery in endometriosis patients where fertility is an issue to prevent adhesions.

Peritoneal changes seem to have an impact on the adhesion and invasion of endometriosis cells, for example, different expression of peritoneal mesothelial adhesion factors including the loss of tight junctions [40]. Tissue remodeling is triggered by matrix-metalloproteinases (MMPs) and the vascular endothelial growth factor (VEGF), which were found differentially expressed in patients with endometriosis [41, 42]. Peritoneal fluid enriched with growth factors and inflammatory regulators also influence tissue remodeling and invasion, like chemokines (interleukin-1B, RANTES, CCL3) or a monocyte secreted factor called the thymus-expressed cytokine (TECK), which stimulates MMP2 and MMP9 production [29]. Estrogen and progesterone play an important role in the development and maintenance of endometriosis. Although endometriotic cells do not have high proliferative activity, supported by Ki67 studies and cell cycle gene expression, stimulation through steroid hormones along with growth factors promotes steady and slow growth [43–45]. Additionally in a mouse model stimulation with estrogen led to an increase of MMP2 and promoted ectopic implantation of endometrial tissue [46]. Interestingly, tissue remodeling is also stimulated after surgical interventions and during wound healing [47–50].

Although incomplete surgical removal of the lesion appears to correlate with recurrence of endometriosis, the exact mechanisms are still unknown [7]. Therefore, we support the hypothesis that after surgical intervention possible remaining free endometriotic cells or tissue residues could readhere and establish growth. Increased levels of different proteins, like MMPs and VEGF [42], might support not only peritoneal healing, but also implantation of endometriotic cells at or near wound sites. Thus, it may be important to prevent direct contact of endometriotic cells with altered peritoneal regions along with decreasing inflammation at wound sites. Although clinical studies are needed, it seems reasonable that when cellular adhesion is inhibited relapse rates could be lowered. The first step of cellular adherence to a substrate is essential for survival. In this study we could show using cell culture that adherence as well as proliferation and ultimately invasion of eight different cell lines including three Ec-ESC and three Eu-Esc primary cell lines and two ovarian carcinoma cell lines were inhibited by a barrier gel containing polyethylene oxide and sodium carboxymethyl cellulose. Importantly, we could demonstrate direct interaction between the barrier agent and these different cell types, especially since adherence and growth were visibly inhibited around and within the barrier gel drop. Furthermore, blocking cellular adherence onto collagen, a component of the basement membrane, resulted in inhibition of invasion. To our knowledge studies using adhesion barriers relating to endometriosis relapse rates have not yet been conducted; however, there have been various hyaluronan containing barrier gel studies relating to tumors (Table 1). Hyaluronan is a proteoglycan (*β*(1,3)-D-glucuronic acid and *β*(1,4)-N-acetyl-D-glucosamine) and with the use of cellulose represents a *β*1–4-linked D-glucose. For example, treatment of rats with a hyaluronan containing barrier along with colorectal cell lines expressing different amounts of the CD44 cell surface marker, a ligand for hyaluronan, increased the tumor nodule count* in vivo* within the peritoneal cavity and tumor proliferation* in vitro* [24]. It was concluded that the presence of hyaluronan caused tumor stimulation in a rat model. In another study, using survival as the outcome, no differences were noted in nude mice following peritoneal injection of a CD44 highly expressing colorectal cell line along with a hyaluronan containing barrier [25]. On the other hand, significant inhibition of adhesion of a colon carcinoma cell line to mesothelial cells occurred* in vitro* when a solution of sodium hyaluronate in phosphate-buffered saline was used; however no significant effect was noted in rats [26]. Regarding an* in vitro* study using cocultures of four ovarian carcinoma cell lines (UCI-101, UCI-107, OC-222, and OVCAR-3) with mesothelial cells demonstrated increased cancer cell motility toward mesothelial cells, which was abolished by hyaluronidase [27]. Importantly, both mesothelial and ovarian carcinoma cells produced up to two times more hyaluronan compared to mesothelial cells alone. In two retrospective short term studies involving colorectal [51] or gynecological cancer patients [28] treatment with or without Seprafilm showed no statistical significance in disease-free survival after 1-2 years or 2.1 years, respectively.

## 5. Conclusions

The molecular mechanism, which leads to recurrence of endometriosis, needs to be further investigated. Our findings suggest that specific barrier agents could be important in preventing recurrence of endometriosis as well as providing an option for ovarian carcinoma patients during surgery. It will be important to further investigate different antiadhesion barrier gel substances to determine their effects on adhesion, proliferation, and invasion of endometriosis cells and ovarian carcinoma cells* in vitro* as well as* in vivo* to determine their significance.

## Figures and Tables

**Table 1:** Liquid antiadhesion barriers, characteristics, and overview of published studies [11, 12, 14, 23–25, 27, 50–59] *in vitro* and *in vivo*.

Name	Active chemical	Company	Intervention	Study	Outcome	Reference
Hyalobarrier	Hyaluronic acid cross-linked polysaccharide	Fidia Adv. Biopolymers	Adhesion	Rabbit	Significant adhesion reduction	[50]
			Myoma	Human	Nonsignificant adhesion reduction	[51]
			Adhesion	Rabbit	Significant adhesion reduction	[52]

Hyalobarrier	0.4% hyaluronic acid cross-linked polysaccharide	Baxter GmbH	Adhesion	Wistar rat	Significant adhesion reduction	[53]

Seprafilm	0.4% hyaluronic acid, carboxymethyl cellulose	Genzyme Corp.	Colorectal cell line HT29	Nude mice	No significance between controls versus treated nude mice	[24]
			Adhesion	Wistar rat	20% were adhesion-free	[54]
			Ovarian cancer, retrospective study, colorectal cancer	Human Human	Significant adhesion reduction Nonsignificant 1- and 2-year disease-free survival rates, no treatment versus treatment	[14]
			Cancers (ovary, fallopian tube, and peritoneum) retrospective study	Human	Nonsignificant 2.1-year disease-free survival rate, no treatment versus treatment	[27, 49]

Sepracoat	0.4% hyaluronic acid, Na^+^-saline	Genzyme Corp.	Colon carcinoma cell line CC-531	*In vitro *	Inhibited tumor cell adhesion on mesothelial cells	[25]
				Rat	No significant effect on tumor growth	[25]

(Pure substance)	Sodium hyaluronate	Pharmacia	Various colorectal carcinoma cell lines CD44+	Rat	Significantly increased tumor nodules, peritoneal cavity	[23]
				*In vitro *	Significant tumor cell proliferation and motility induction	[23]

Adept	4% icodextrin (*α*1-4-linked dextrin)	Shire GmbH	Adhesion	Wistar rat	Significant adhesion reduction	[53]
			Adhesion	Wistar rat	Adhesion-free incidence 0%	[54]

Oxiplex/AP gel	Polyethylene oxide carboxymethyl cellulose	FzioMed, Inc.	Adhesion including patients with endometriosis	Human	Marked adhesion reduction	[11]
				Human	Significant adhesion reduction	[12]

Intercoat	Polyethylene oxide, sodium carboxymethyl cellulose	Ethicon, Inc.	Adhesion	Balb/c mice	Significant adhesion reduction	[55]
			Adhesion	Wistar rat	20% were adhesion-free	[54]
			Adhesion	Human	Significant adhesion reduction	[56]
			Adhesion	Human	Not significant	[57]
			Adhesion, proliferation, and invasion	*In vitro*: primary Ec-ESC, Eu-ESC, ovarian carcinoma cell lines	Significant reduction of adherence, proliferation, and invasion	This study

## References

[1] Larosa M., Facchini F., Pozzoli G., Leone M., Grande M., Monica B. (2010). Endometriosis: aetiopathogenetic basis. *Urologia*.

[2] Burghaus S., Klingsiek P., Fasching P. A. (2011). Risk factors for endometriosis in a German case-control study. *Geburtshilfe und Frauenheilkunde*.

[3] Renner S. P., Strick R., Oppelt P. (2006). Evaluation of clinical parameters and estrogen receptor alpha gene polymorphisms for patients with endometriosis. *Reproduction*.

[4] Olive D. L., Schwartz L. B. (1993). Endometriosis. *The New England Journal of Medicine*.

[5] Falcone T., Lebovic D. I. (2011). Clinical management of endometriosis. *Obstetrics and Gynecology*.

[6] Renner S. P., Rix S., Boosz A. (2010). Preoperative pain and recurrence risk in patients with peritoneal endometriosis. *Gynecological Endocrinology*.

[7] Taylor E., Williams C. (2010). Surgical treatment of endometriosis: location and patterns of disease at reoperation. *Fertility and Sterility*.

[8] Koninckx P. R., Ussia A., Adamyan L., Wattiez A., Donnez J. (2012). Deep endometriosis: definition, diagnosis, and treatment. *Fertility and Sterility*.

[9] Diamond M. P., Freeman M. L. (2001). Clinical implications of postsurgical adhesions. *Human Reproduction Update*.

[10] Liakakos T., Thomakos N., Fine P. M., Dervenis C., Young R. L. (2001). Peritoneal adhesions: Etiology, pathophysiology, and clinical significance - Recent advances in prevention and management. *Digestive Surgery*.

[11] Vrijland W. W., Jeekel J., van Geldorp H. J., Swank D. J., Bonjer H. J. (2003). Abdominal adhesions: intestinal obstruction, pain, and infertility. *Surgical Endoscopy and Other Interventional Techniques*.

[12] Young P., Johns A., Templeman C. (2005). Reduction of postoperative adhesions after laparoscopic gynecological surgery with Oxiplex/AP Gel: a pilot study. *Fertility and Sterility*.

[13] Lundorff P., Donnez J., Korell M., Audebert A. J. M., Block K., diZerega G. S. (2005). Clinical evaluation of a viscoelastic gel for reduction of adhesions following gynaecological surgery by laparoscopy in Europe. *Human Reproduction*.

[14] Robertson D., Lefebvre G., Leyland N. (2010). Adhesion prevention in gynaecological surgery. *Journal of Obstetrics and Gynaecology Canada*.

[15] Bristow R. E., Montz F. J. (2005). Prevention of adhesion formation after radical oophorectomy using a sodium hyaluronate-carboxymethylcellulose (HA-CMC) barrier. *Gynecologic Oncology*.

[16] Ahmad G., Duffy J. M., Farquhar C. (2008). Barrier agents for adhesion prevention after gynaecological surgery. *Cochrane Database of Systematic Reviews*.

[17] Pellicano M., Guida M., Bramante S. (2005). Reproductive outcome after autocrosslinked hyaluronic acid gel application in infertile patients who underwent laparoscopic myomectomy. *Fertility and Sterility*.

[18] Johns D. B., Keyport G. M., Hoehler F., DiZerega G. S. (2001). Reduction of postsurgical adhesions with intergel adhesion prevention solution: a multicenter study of safety and efficacy after conservative gynecologic surgery. *Fertility and Sterility*.

[19] (1988). The cervical cap. *The Medical Letter on Drugs and Therapeutics*.

[20] Laschke M. W., Rudzitis-Auth J., Menger M. D. (2013). Regional treatment with liquid barrier agents: a novel therapeutic option for intraperitoneal endometriosis?. *Medical Hypotheses*.

[21] Kyama C. M., Mihalyi A., Simsa P. (2009). Role of cytokines in the endometrial-peritoneal cross-talk and development of endometriosis. *Frontiers in Bioscience*.

[22] Toole B. P., Wight T. N., Tammi M. I. (2002). Hyaluronan-cell interactions in cancer and vascular disease. *Journal of Biological Chemistry*.

[23] Götte M., Yip G. W. (2006). Heparanase, hyaluronan, and CD44 in cancers: a breast carcinoma perspective. *Cancer Research*.

[24] Tan B., Wang J. H., Wu Q. D., Kirwan W. O., Redmond H. P. (2001). Sodium hyaluronate enhances colorectal tumour cell metastatic potential in vitro and in vivo. *British Journal of Surgery*.

[25] Pucciarelli S., Codello L., Rosato A., del Bianco P., Vecchiato G., Lise M. (2003). Effect of antiadhesive agents on peritoneal carcinomatosis in an experimental model. *British Journal of Surgery*.

[26] Haverlag R., van Rossen M. E. E., van den Tol M. P., Bonthuis F., Marquet R. L., Jeekel J. (1999). Hyaluronate-based coating solution for prevention of surgical adhesions has no major effect on adhesion and growth of intraperitoneal tumour cells. *European Journal of Surgery*.

[27] Carpenter P. M., Dao A. V. (2003). The role of hyaluronan in mesothelium-induced motility of ovarian carcinoma cells. *Anticancer Research*.

[28] Tan A., Argenta P., Ramirez R., Bliss R., Geller M. (2009). The use of sodium hyaluronate-carboxymethylcellulose (HA-CMC) barrier in gynecologic malignancies: a retrospective review of outcomes. *Annals of Surgical Oncology*.

[29] Weimar C. H. E., Macklon N. S., Post Uiterweer E. D., Brosens J. J., Gellersen B. (2013). The motile and invasive capacity of human endometrial stromal cells: implications for normal and impaired reproductive function. *Human Reproduction Update*.

[30] Sampson J. A. (1927). Metastatic or embolic endometriosis, due to the menstrual dissemination of endometrial tissue into the venous circulation. *The American Journal of Pathology*.

[31] Gilks C. B. (2010). Molecular abnormalities in ovarian cancer subtypes other than high-grade serous carcinoma. *Journal of Oncology*.

[32] Pearce C. L., Templeman C., Rossing M. A. (2012). Association between endometriosis and risk of histological subtypes of ovarian cancer: a pooled analysis of case-control studies. *The Lancet Oncology*.

[33] Zeitvogel A., Baumann R., Starzinski-Powitz A. (2001). Identification of an invasive, N-cadherin-expressing epithelial cell type in endometriosis using a new cell culture model. *The American Journal of Pathology*.

[34] Ornek T., Fadiel A., Tan O., Naftolin F., Arici A. (2008). Regulation and activation of ezrin protein in endometriosis. *Human Reproduction*.

[35] Kao A.-P., Wang K.-H., Chang C.-C. (2011). Comparative study of human eutopic and ectopic endometrial mesenchymal stem cells and the development of an in vivo endometriotic invasion model. *Fertility and Sterility*.

[36] Yotova I., Quan P., Gaba A. (2012). Raf-1 levels determine the migration rate of primary endometrial stromal cells of patients with endometriosis. *Journal of Cellular and Molecular Medicine*.

[37] Santamaria X., Massasa E. E., Taylor H. S. (2012). Migration of cells from experimental endometriosis to the uterine endometrium. *Endocrinology*.

[38] Wacker I., Sachs M., Knaup K. (2009). Key role for activin B in cellular transformation after loss of the von Hippel-Lindau tumor suppressor. *Molecular and Cellular Biology*.

[39] Parker M. C., Ellis H., Moran B. J. (2001). Postoperative adhesions: ten-year follow-up of 12,584 patients undergoing lower abdominal surgery. *Diseases of the Colon and Rectum*.

[40] Young V. J., Brown J. K., Saunders P. T. K., Horne A. W. (2013). The role of the peritoneum in the pathogenesis of endometriosis. *Human Reproduction Update*.

[41] MacHado D. E., Berardo P. T., Palmero C. Y., Nasciutti L. E. (2010). Higher expression of vascular endothelial growth factor (VEGF) and its receptor VEGFR-2 (Flk-1) and metalloproteinase-9 (MMP-9) in a rat model of peritoneal endometriosis is similar to cancer diseases. *Journal of Experimental and Clinical Cancer Research*.

[42] Malvezzi H., Aguiar V. G., de Paz C. C. P., Tanus-Santos J. E., Penna I. A. D. A., Navarro P. A. (2013). Increased circulating MMP-2 levels in infertile patients with moderate and severe pelvic endometriosis. *Reproductive Sciences*.

[43] Sohler F., Sommer A., Wachter D. L. (2013). Tissue remodeling and nonendometrium-like menstrual cycling are hallmarks of peritoneal endometriosis lesions. *Reproductive Sciences*.

[44] Calcagno A., Grassi T., Mariuzzi L. (2011). Expression patterns of Aurora A and B kinases, Ki-67 and the estrogen and progesterone receptors determined using an endometriosis tissue microarray model. *Human Reproduction*.

[45] Scotti S., Regidor P.-A., Schindler A. E., Winterhager E. (2000). Reduced proliferation and cell adhesion in endometriosis. *Molecular Human Reproduction*.

[46] Wang J., Ma X. (2012). Effects of estrogen and progestin on expression of MMP-2 and TIMP-2 in a nude mouse model of endometriosis. *Clinical and Experimental Obstetrics and Gynecology*.

[47] Cheong Y. C., Laird S. M., Li T. C., Shelton J. B., Ledger W. L., Cooke I. D. (2001). Peritoneal healing and adhesion formation/reformation. *Human Reproduction Update*.

[48] Haney A. F. (2000). Identification of macrophages at the site of peritoneal injury: evidence supporting a direct role for peritoneal macrophages in healing injured peritoneum. *Fertility and Sterility*.

[49] Bittinger F., Schepp C., Brochhausen C. (1999). Remodeling of peritoneal-like structures by mesothelial cells: its role in peritoneal healing. *Journal of Surgical Research*.

[50] Yung S., Davies M. (1998). Response of the human peritoneal mesothelial cell to injury: an in vitro model of peritoneal wound healing. *Kidney International*.

[51] Oikonomakis I., Wexner S. D., Gervaz P., You S.-Y., Secic M., Giamundo P. (2002). Seprafilm: a retrospective preliminary evaluation of the impact on short-term oncologic outcome in colorectal cancer. *Diseases of the Colon and Rectum*.

[52] de laco P. A., Stefanetti M., Pressato D. (1998). A novel hyaluronan-based gel in laparoscopic adhesion prevention: preclinical evaluation in an animal model. *Fertility and Sterility*.

[53] Mais V., Bracco G. L., Litta P., Gargiulo T., Melis G. B. (2006). Reduction of postoperative adhesions with an auto-crosslinked hyaluronan gel in gynaecological laparoscopic surgery: a blinded, controlled, randomized, multicentre study. *Human Reproduction*.

[54] Belluco C., Meggiolaro F., Pressato D. (2001). Prevention of postsurgical adhesions with an autocrosslinked hyaluronan derivative gel. *Journal of Surgical Research*.

[55] Wallwiener M., Brucker S., Hierlemann H., Brochhausen C., Solomayer E., Wallwiener C. (2006). Innovative barriers for peritoneal adhesion prevention: liquid or solid? A rat uterine horn model. *Fertility and Sterility*.

[56] Rajab T. K., Wallwiener M., Planck C., Brochhausen C., Kraemer B., Wallwiener C. W. (2010). A direct comparison of seprafilm, adept, intercoat, and spraygel for adhesion prophylaxis. *Journal of Surgical Research*.

[57] Schonman R., Corona R., Bastidas A., de Cicco C., Mailova K., Koninckx P. R. (2009). Intercoat gel (oxiplex): efficacy, safety, and tissue response in a laparoscopic mouse model. *Journal of Minimally Invasive Gynecology*.

[58] Di Spiezio Sardo A., Spinelli M., Bramante S. (2011). Efficacy of a polyethylene oxide-sodium carboxymethylcellulose gel in prevention of intrauterine adhesions after hysteroscopic surgery. *Journal of Minimally Invasive Gynecology*.

[59] Fuchs N., Smorgick N., Ben Ami I. (2014). Intercoat (Oxiplex/AP gel) for preventing intrauterine adhesions after operative hysteroscopy for suspected retained products of conception: double-blind, prospective, randomized pilot study. *Journal of Minimally Invasive Gynecology*.

